# National Association of Dental Faculties

**Published:** 1899-09

**Authors:** 


### NATIONAL ASSOCIATION OF DENTAL FACULTIES.

The annual meeting of this body is always looked forward to by its members with great interest, and it is rare to find any college, however distant, but has its representative present. This was peculiarly true of the recent session held at Niagara. This convened on the morning of July 28 with a full attendance.

It was not anticipated that this meeting would result in any changes in the general order adopted last year, and, in view of this, a short session was anticipated. The results demonstrated that the unexpected happens, for this body did not reach a final adjournment until August 3, the longest period in its history.

This prolongation of the sessions was not due to any extraordinary amount of business, but to the necessity for frequent adjournments to allow committees to complete their work, and especially that of the Conference Committee appointed to meet with a similar committee appointed by the National Association of Dental Examiners.

The feeling in the Faculties at the opening of the first session was one of extreme bitterness towards the National Association of Examiners, and a disposition was manifested in the Faculties to defend the legal rights of the colleges to the last extremity. This feeling was evidently reflected upon the other National body, and resulted in an official request that a Committee of Conference be appointed. This was finally acceded to, and after repeated sessions of the joint committee an harmonious conclusion was reached and finally adopted by the two national organizations. This was not accomplished without considerable friction in both associations, resulting, on the part of the Examiners, in the withdrawal of the New Jersey State Board.

The conclusions arrived at places the National Association of Dental Examiners and the National Association of Dental Faculties upon the same educational basis. The former accepts the position of the latter and agrees, as far as lies in its power, to withdraw all suits now pending and, as far as possible, retract charges of disreputability made against several colleges. This conclusion of the two organizations was a most satisfactory solution of a difficult problem, and if no other good was accomplished by the meetings at Niagara this alone compensated for the expenditure of time, strength, and money, for it ends, for the time being, serious litigation that promised disastrous results to the cause of dental education. If now the States not represented in the National Association of Dental Examiners will adopt the same liberal policy, then may speedily come a solution of many vexed questions. If, however, they continue the old antagonistic methods there will be but one course for the colleges to pursue,—that of following the matter through to the highest courts. It is hoped wise counsels will prevail.

The necessity for a united body at this period in the history of dental education must be apparent to all observant minds. To accomplish this all methods and measures that tend to disintegration should be determinedly opposed and be regarded as a menace to the life of the organization. For the first time the political caucus was introduced at Omaha in the Association of Faculties, and this year this objectionable method was repeated at Niagara. This cannot go on without ending in the destruction of this organization. The universities and higher medical schools connected with it have sacrificed much in their efforts to strengthen the weaker schools, but there is a limit to this, and if the secret plans evidently adopted at the recent meeting are to be continued, the smaller schools may find themselves struggling unsupported in a maze of difficulties. The universities of this country are not in this work for office honors, and the future may not find them remaining in an association where the methods of the politician outrank, in importance, the work of the scholar. The true position is for all the schools to aim at a standard beyond criticism and by methods in which no flaw can be discovered.

